# Residual neuromuscular block in the postanesthesia care unit: incidence, risk factors, and effect of neuromuscular monitoring and reversal agents

**DOI:** 10.55730/1300-0144.5507

**Published:** 2022-07-24

**Authors:** Nazlı Bahar ÖZBEY, Taner ABDULLAH, Özlem DELİGÖZ

**Affiliations:** 1 Department of Anesthesiology, Başaksehir Çam and Sakura City Hospital, University of Health Sciences, İstanbul, Turkey; 2 Department of Anesthesiology and Reanimation, Haydarpaşa Numune Training and Research Hospital, University of Health Sciences, İstanbul, Turkey

**Keywords:** Residual neuromuscular block, neuromuscular monitoring, neostigmine, sugammadex

## Abstract

**Background/aim:**

The aim of this study was to determine the incidence of residual neuromuscular block (RNMB) in a tertiary care hospital. Secondary goals were to examine the characteristics of the use of intraoperative neuromuscular monitoring (NMM) and different reversal agents by the attending anesthesiologists, and to determine the factors related to the patient and perioperative processes on the development of RNMB.

**Materials and methods:**

The patients’ arrival time at the postanesthesia care unit was accepted as point zero (T0). The acceleromyography of the patients’ adductor pollicis muscle was monitored for NMM. Train of four ratios (TOFRs) were recorded at 0, 10, 20, and 30 min. A TOFR < 0.9 was defined as RNMB. Patients’ demographic and perioperative data were also recorded.

**Results:**

A total of 216 patients completed the study. RNMB was observed in 47 patients (21.8%). Seventy-eight patients (36%) were followed up with NMM. Neostigmine and sugammadex were used in 174 (80.5%) and 42 (19.5%) patients, respectively, and they were both underdosed (21.2 ± 3.0 mcg/kg and 1.5 ± 0.7 mg/kg, respectively). Use of neostigmine and absence of NMM were risk factors for RNMB (p: 0.01 and 0.001, respectively) along with the number of additional doses (>1 doses, p ≤ 0.02) and the timing of the last dose of rocuronium (<88 min, p ≤ 0.01). None of the patients who received both NMM and sugammadex experienced RNMB.

**Conclusion:**

The RNMB incidence was found to be 21.8%. The main reasons of it were the lack of intraoperative NMM and inappropriate use of reversal agents. Despite strong recommendations, the use of NMM is still insufficient and reversal agents are still underdosed.

## 1. Introduction

Neuromuscular blocker drugs (NMBDs) are frequently used during anesthesia to facilitate tracheal intubation, maintain mechanical ventilation, and facilitate surgeries. Owing to an insufficient reversal of these agents, residual neuromuscular block (RNMB) may be observed during the postoperative period. RNMB is defined as a train-of-four ratio (TOFR) < 0.9 [1].

RNMB has been reported in 13% to 88% of patients in the postanesthesia care unit (PACU) [1–6]. RNMB in the early postoperative period is related to delayed tracheal extubation, acute respiratory events (hypoxemia and airway obstruction), muscle weakness, prolonged PACU stay, and increased risk of postoperative pulmonary complications [7, 8]. Therefore, the most recent guideline on the use of NMBDs and reversal agents, published in 2020, recommends qualitative or quantitative neuromuscular evaluation before administration of a reversal agent. It is recommended that, if the monitored TOF count is 4, then neostigmine (40 mcg/kg) + atropine (0.02 mg/kg) can be administered. When sugammadex is used, the minimum dose suggested is 2 mg/kg in accordance with the neuromuscular monitoring (NMM) data [9].

Studies published before the 2020 guideline showed that very few clinicians acknowledged the necessity of NMM and routinely performed it in their cases [10]. Moreover, reversal agents are frequently underdosed in light of recent recommendations [8,11]. To the best of our knowledge, no observational studies have examined the use of NMM and reversal agents after the aforementioned guideline was published.

The primary objective of this study was to determine the incidence of RNMB in a tertiary care hospital with available NMM and the use of both reversal agents. The secondary goals were to examine the characteristics of the use of intraoperative NMM and different reversal agents by the attending anesthesiologists, and to determine the factors related to the patient and perioperative processes on the development of RNMB in a real-life clinical setting.

## 2. Materials and Methods

This prospective observational study was initiated with the approval of the Haydarpaşa Numune Training and Research Hospital Clinical Research Ethics Committee’s application date 24.02.2020 and decision number HNEAH-KAEK 2020/KK/26. Written informed consent was obtained from all patients.

### 2.1. Patients

Patients who were 18–80 years of age, in the ASA I-III risk group, and scheduled to undergo orotracheal intubation using NMBDs under general anesthesia were included in this study.

Patients with neuromuscular disease, renal failure (GFR <60), hepatic failure (INR >1.5), history of aminoglycoside antibiotic use, pregnancy, planned postoperative follow-up in the intensive care unit, unplanned intensive care unit admission, or any diversion from general anesthesia with orotracheal intubation (e.g., anesthesia plan changed to the use of supraglottic airway device or regional techniques) were excluded from this study.

### 2.2. Protocol

Patient premedication and intraoperative anesthesia applications were selected by the anesthesiologist who monitored the patient. Intraoperative NMM and reversal agent selection were also performed by the relevant anesthesiologist. In line with the observational study design, the doses of NMBDs and reversal agents were not standardized.

The patient arrival time at the PACU was designated as point zero (T0). In addition to the vital signs, acceleromyography (TOF-Watch®-SX Monitor, Organon Teknika, Dublin, Ireland) of the patient’s adductor pollicis muscle was monitored by a trained PACU nurse who was blinded to the study. The stimulation current was set at 50 mA. The average of two consecutive measurements made at a frequency of 2 Hz, with four pulses of 0.2 ms duration separated by 15 s, was recorded. If the difference between measurements was more than 10%, additional TOFR measurements (up to four values) were performed, and the average of the two closest measurements was taken. The patient’s arterial oxygen saturation (SpO_2_) was measured using a pulse oximeter. Heart rate (HR), systolic and diastolic blood pressure, and TOFR values were recorded at 0, 10, 20, and 30 min (T0, T1, T2, and T3, respectively). A TOFR < 0.9 was defined as RNMB. In the presence of RNMB, additional reversal agents and their doses, as considered necessary by the anesthesiologist, were documented.

Following the completion of the protocol, patients’ demographic and perioperative data were recorded from their pre- and intraoperative evaluations as follows:

- Height, weight, body mass index, age, sex, comorbidities, ASA score, drugs used, doses and timings of agents used in induction and maintenance of anesthesia, type of NMBD used, induction dose and total dose, number of additional doses, and timing.
- Whether NMM was performed on the patient, the reversal agent administered at the end of the operation, dose, and timing.
- Duration of anesthesia: The time between the start of induction and the decision to transfer to the PACU.
- Reversal-PACU: Time between the first administration of the reversal agent at the end of surgery and T0 in the PACU.
- NMBD-PACU: Time between the last NMBD administration and T0 in the PACU.

### 2.3. Statistical Analyses

Assuming that the incidence of RNMB would be at most 40%, the sample size, with alpha 0.05 and beta 0.1, was calculated as 210 patients. With this sample size, the predicted incidence could be estimated with a margin of error of < 7% at the 95% confidence interval.

Categorical variables were compared using the chi-square test and are expressed as numbers and percentages. Subgroups were compared using Bonferroni correction. The distribution of continuous variables was evaluated using the Shapiro–Wilk test. Normally distributed variables were compared using the Student’s t-test and are expressed as means and standard deviations. Continuous variables without normal distribution were compared using the Mann–Whitney U test and are expressed as medians and 25th–75th percentiles. The binary logistic regression test was used to evaluate the relationship between risk factors and RNMB. To categorize continuous variables in the regression analysis, receiver operating characteristic (ROC) curve analysis was performed to determine the optimal cutoff point using Youden’s index (sensitivity + specificity − 1) and points at which the specificity and sensitivity values were 90%. Four groups were formed based on these three points. In all statistical analyses, statistical significance was set at p < 0.05. Statistical analyses were performed using SPSS for Windows, version 21.0 (SPSS Inc., Chicago, IL, USA) or MedCalc, version 16.1 (MedCalc Software Ltd, Ostend, Belgium), as appropriate.

## 3. Results

A total of 239 patients were included in the study, 216 of whom completed the protocol (Figure). Rocuronium was used as the neuromuscular blocking agent in all patients, and all patients received a reversal agent at the end of surgery.

According to the initial TOFR values measured in the recovery room, RNMB was observed in 47 patients (21.8%). Based on the development of RNMB, the patients were allocated into two groups (RNMB and no-RNMB) and compared. Data regarding the patients’ demographic characteristics, comorbidities, surgical characteristics, volatile agents used, and duration of anesthesia are given in Table 1. Based on the first vital signs obtained in the PACU, there was a significant difference in SpO_2_ and HR values in favor of the no-RNMB group (p = 0.007 and p < 0.001, respectively) (Table 2).

Seventy-eight patients (36%) were followed with acceleromyography for NMM during the intraoperative period. RNMB developed less frequently in these patients compared with those who were not monitored (11.5% vs. 27.5%, p = 0.01). This difference, which was still observed at T1 and T2, vanished at T3 (p values: 0.02, 0.05, and 0.55 for the aforementioned time points, respectively) (Table 3).

In 174 (80.5%) patients, neostigmine/atropine was administered; in 42 patients (19.5%), sugammadex was administered. In the sugammadex group, 35 of 42 patients received a dose < 2 mg/kg. A lower incidence of RNMB was observed in patients who received sugammadex compared with those who received neostigmine (4.8% vs. 25.9%, p = 0.006). The rocuronium doses administered during induction and throughout the surgery, the total number of additional NMBD doses, NMBD-PACU duration, reversal agents administered and their doses, and reversal-PACU duration in the RNMB and no-RNMB groups are shown in Table 4.

Patients were allocated into four groups according to whether they were followed with NMM and the reversal agent used:

- NMM was applied, neostigmine was used (N+); n = 49.
- No NMM, neostigmine was used (N−); n = 125.
- NMM was applied, sugammadex was used (S+); n = 29.
- No NMM, sugammadex was used (S−); n = 13.

The incidences of RNMB in these groups were 18%, 28%, 0%, and 15%, respectively (p = 0.006). In the subgroup analysis, only the difference between the S+ and N− groups was statistically significant (p = 0.001).

Three patients in the N− group had TOFR values of 0.65, 0.63, and 0.67, and an additional 1-mg dose of neostigmine was administered to these patients. When the T3 TOFR values were examined, the TOFR value was > 0.9 in one patient, and RNMB persisted in the other two (TOFR: 0.85, 0.82). Except for these three patients, none received any additional doses, as the attending clinicians preferred to follow the patients in their existing state instead of intervening with additional doses.

Eight patients had TOFR values of 0.70–0.79; all were in the N− group. The attending anesthesiologists also preferred to follow these patients in their existing state without intervention. They were all under RNMB at T1, while two had recovered at T2, and all had TOFRs > 0.9 at T3.

The TOFR values of two patients in the S− group with RNMB were 0.86 and 0.82 at T0. The TOFR values of these two patients were > 0.9 at T1. The doses of sugammadex administered to these patients were 1.1 mg/kg and 3.2 mg/kg, respectively. The duration of anesthesia and total rocuronium dose of the latter patient were 292 min and 1.4 mg/kg, respectively. None of the patients in the S+ group experienced RNMB despite the low doses used for sugammadex.

The following variables were included in the binary logistic regression analysis to evaluate the relationship between these variables and the occurrence of RNMB: the number of additional NMBD doses (reference value: zero additional doses), whether intraoperative NMM was performed (reference value: NMM was performed), reversal agent administered (reference value: sugammadex used), and NMBD-PACU (reference value: 0–50 min). The NMBD-PACU was categorized as 0–50 min, 51–87 min, 88–144 min, and 145+ min according to the points on the ROC curve defined in the Materials and Methods section. The probability ratios, confidence intervals, and p values of the related variables are presented in Table 5. A NMBD-PACU value < 88 min was significantly related to increased risk for RNMB (OR ≥ 7.6; p ≤ 0.001).

## 4. Discussion

In this study, RNMB was detected in 21.8% of the patients. In studies using the TOFR < 0.9 threshold, the incidence of RNMB varies greatly from 13% to 88% [1–6]. This discrepancy between the reported values may be due to substantial differences in study methodology and protocols, such as the application of intraoperative NMM, type of NMM equipment used, waiting for spontaneous recovery vs. intervention with a reversal agent, and type and dose of the reversal agent used.

NMM was applied to 78 (36%) of the patients, and the incidence of RNMB was higher in non-monitored patients (27.5% vs. 11.5%, p = 0.01; OR: 2.65, p = 0.03). Similarly, in the study conducted by Murphy et al., the incidence of RNMB was lower in patients who underwent intraoperative NMM (14.5% vs. 50.0%, p < 0.0001) [12]. Baillard et al. showed that RNMB incidence could decrease from 62% to 3% after training for the use of NMM and proper use of reversal agents [13].

The 21.8% incidence of RNMB despite NMM application in our study can be explained by the dose, timing, and duration to peak effect of the reversal agents administered. The neostigmine doses administered in our study (21.2 ± 3.0 mcg/kg) were below the recommended 40 mcg/kg [9]. The tendency of anesthesiologists to administer low doses of neostigmine may be because neostigmine doses of ≥ 2.5 mg can increase the incidence of nausea, vomiting, and other anticholinergic effects [14, 15]. In addition, Caldwell et al. reported that when neostigmine is administered to a patient who has nearly or completely recovered from a neuromuscular block, it may decrease TOFR and cause muscle weakness as a result of prolonged paradoxical neuromuscular block [16]. In a study of the duration to peak effect of neostigmine, Della Rocca et al. revealed that the times to reach TOFR ≥ 0.9 for patients with a superficial block (TOF count: 2) and deep block are 7.8 min (0.5–37.8 min) and 20.6 min (1.8–41.3 min), respectively [11]. Based on these data, we can conclude that anesthesiologists prefer to wait for spontaneous recovery instead of administering an additional dose in cases of asymptomatic RNMB. Thus, despite the similar neostigmine doses in patients with and without RNMB, the longer reversal-PACU time (13.6 ± 3.6 min vs. 11.7 ± 3.5 min, p = 0.001) in the RNMB group seems to be due to the longer recovery time of these patients. We can assume that these patients were inadequately reversed, spent more time in the operating room due to struggles during recovery, and were still under RNMB when arriving at the PACU.

In our study, sugammadex was used in 42 (19.4%) patients. The attending anesthesiologists preferred a sugammadex dose less than the recommended 2 mg/kg in 35 (83%) of them. Schaller et al. revealed that a sugammadex dose of 0.22 mg/kg is sufficient to reverse a shallow block, defined as TOFR 0.5 [17]. In addition, researchers have found that when the TOF count is 2–4 (moderate block), a sugammadex dose of 1 mg/kg can be safely used for reversal [18]. Considering these findings, attending anesthesiologists might have preferred lower doses of sugammadex based on NMM results or their clinical experience. Additionally, the high cost of sugammadex, when compared to that of neostigmine might have been a reason for underdosing.

We observed RNMB in 2 (4.8%) patients who received sugammadex. One was underdosed (1.1 mg/kg), while the other received a dose expected to be sufficient (3.2 mg/kg). Neither of these patients underwent NMM. RNMB was not observed in any patient who underwent NMM and received sugammadex. Kotake et al. found a 9.4% incidence of RNMB in patients who did not undergo NMM and were administered sugammadex [19]. Understandably, although the use of sugammadex reduces RNMB, it cannot guarantee safe extubation in the absence of NMM [19, 20].

Factors that increase the risk of RNMB include the number of doses and the timing of the last dose of NMBD [21]. In our study, the probability of developing RNMB gradually increased as the NMBD-PACU duration shortened and the number of additional NMBD doses increased, starting from the second additional dose (Table 3). Our results indicate that increased risk for RNMB may last up to 88 min after the last administered rocuronium dose. Similarly, Naguib et al. showed that high and additional doses of NMBD administration and short NMBD-PACU duration increased the cumulative effect and risk of RNMB [22].

We believe that this study contributes to the literature in several ways. To the best of our knowledge, this is the first study evaluating attending anesthesiologists’ preferences regarding the dosing of both neostigmine and sugammadex and the use of NMM after the last guideline published in 2020 [9]. It is interesting to note that specialists continue to prefer lower neostigmine doses despite the recommendations. Further studies are needed to clarify the safety of neostigmine doses > 40 mcg/kg. Additionally, our study revealed that when NMM is applied, RNMB is not expected even when the dose of sugammadex is lowered (<2 mg/kg). Lastly, our study revealed a significantly increased risk for RNMB for 88 min after the last administered dose of rocuronium. This is the first study to reveal such a duration for rocuronium as an independent risk factor for RNMB.

This study has certain limitations. Firstly, patients with an ASA score > 3 and those who required intensive care unit admission in the postoperative period, were not included in the study. The response of these patients to the agents used may lead to different results. Secondly, the intraoperative period, interventions for RNMB diagnosis, agents used, and doses were not standardized. Different results may be obtained in a standardized study. However, our approach reflected real-life situations; therefore, the findings are valuable. Thirdly, postoperative TOFR measurements were performed using a nonprecalibrated TOF-Watch®-SX monitor. As the monitor was set up when the patient arrived in the recovery room, it could not be calibrated by taking supramaximal flow values before NMBD administration. However, this monitor’s basic configuration provides adequate sensitivity for most adult patients; this device has been used without calibration in many studies in which NMM was performed in the recovery room [4, 8, 20)].

## 5. Conclusions

In our study, the RNMB incidence was found to be 21.8% in a tertiary care hospital with available NMM and the use of the reversal agents, neostigmine and sugammadex. Despite strong recommendations, the use of NMM is still insufficient and reversal agents are still underdosed. Lack of intraoperative quantitative NMM, use of neostigmine instead of sugammadex, multiple additional doses, and administration less than 88 min after the last dose of NMBD were identified as risk factors for RNMB. Further efforts should aim towards increasing awareness of RNMB and promoting the routine and proper use of NMM and reversal drugs.

## Figures and Tables

**Figure:**
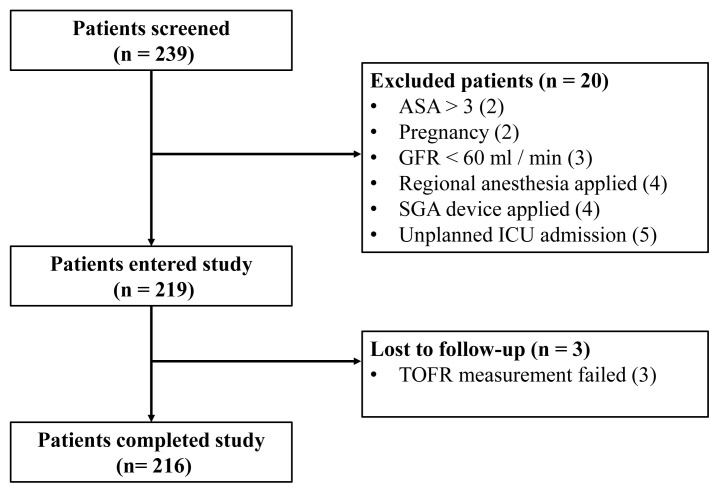
Study flow-chart.

**Table 1:** Characteristics of patients.

	All (216)	RNMB (47)	No-RNMB (169)	p-value
**Sex (M/F)**	110/106	24/23	86/83	0.98
**Age**	50.9 ± 13.2	52 ± 15	51 ± 13	0.52
**BMI (kg/m** **^2^** **)**	27.9 (24.8–31.2)	28.6 (24.6–32.4)	27.7 (24.8–31)	0.67
**ASA Score (1 & 2/3)**	178/38	37/10	141/28	0.51
**Nonsmoker**	153 (71%)	35 (74%)	118 (70%)	0.66
**Comorbidities**				
Hypertension	53 (%25)	12 (%26)	41 (%24)	0.85
Diabetes mellitus	48 (%22)	12 (%26)	36 (%21)	0.55
Coronary artery disease	27 (%13)	4 (%9)	23 (%14)	0.46
Pulmonary diseases	17 (%8)	1 (%2)	16 (%10)	0.13
Malignancy	27 (%13)	9 (%19)	18 (%11)	0.14
Thyroid dysfunctions	22 (%10)	2 (%4)	20 (%12)	0.18
**Duration of anesthesia (min)**	163 ± 65	176 ± 68	159 ± 63	0.10
**Volatile agent**				0.86
Sevoflurane	168 (%78)	37 (%79)	131 (%76)	
Desflurane	48 (%22)	10 (%21)	38 (%24)	
**Surgery group**				0.62
General surgery	113 (%52)	26 (%55)	87 (%51)	
Gynecology	38 (%18)	6 (%13)	32 (%19)	
Urology	28 (%13)	5 (%11)	23 (%14)	
Orthopedic	37 (%17)	10 (%21)	27 (%16)	

Qualitative data are expressed as number and percentage of case, and compared with chi-squared test. Normally distributed data are expressed as mean ± SD, and compared with Student’s t-test. Nonnormally distributed data are expressed as median (25th to 75th percentile), and compared with the Mann–Whitney U test. RNMB: residual neuromuscular block, ASA score: American Society of Anesthesiologists score, BMI: body mass index

**Table 2:** Vital parameters of patients recorded following the arrival at PACU.

	RNMB (47)	No-RNMB (169)	p-value
**SpO2 (%)**	96.74 ± 1.52	97.36 ± 0.96	**0.007**
**Systolic AP (mmHg)**	126.81 ± 23.25	128.62 ± 14.71	0.113
**Diastolic AP (mmHg)**	68.43 ± 11.73	67.22 ± 8.65	0.798
**Mean AP (mmHg)**	86.35 ± 13.07	86.46 ± 9.90	0.371
**Heart rate (1/min)**	79.96 ± 6.08	74.98 ± 8.33	**<0.001**

Data are expressed as mean ± SD, and compared with Student’s t-test. PACU: postoperative anesthesia care unit, RNMB: residual neuromuscular block, SpO2: oxygen saturation of arterial blood measured by pulse oximeter, AP: arterial pressure

**Table 3:** Residual neuromuscular block incidence and train of four ratios according to neuromuscular monitoring application.

	NMM	No-NMM	p-value
**T0-RNMB incidence**	9 (11.5%)	38 (27.5%)	**0.01**
**T1-RNMB incidence**	2 (2.6%)	25 (18.1%)	**0.002**
**T2-RNMB incidence**	0	12 (8.7%)	**0.005**
**T3-RNMB incidence**	0	3 (2.2%)	0.56
**T0-TOF ratio**	0.93 ± 0.04	0.89 ± 0.06	**<0.001**
**T1-TOF ratio**	0.96 ± 0.04	0.93 ± 0.05	**<0.001**
**T2-TOF ratio**	0.99 ± 0.04	0.96 ± 0.04	**<0.001**
**T3-TOF ratio**	1.01 ± 0.04	1.00 ± 0.05	**0.004**

Qualitative data are expressed as number and percentage of case, and compared with chi-squared test. Normally distributed data are expressed as mean ± SD, and compared with Student’s t-test. NMM: neuromuscular monitoring, RNMB: residual neuromuscular block, TOF: train of four

**Table 4:** Perioperative management of neuromuscular junction.

	All (216)	RNMB (47)	No-RNMB (169)	p-value
**Doses of NMBD**				
**Induction dose (mg/kg)**	0.65 ± 0.09	0.65 ± 0.08	0.65 ± 0.10	0.65
**Total dose (mg/kg)**	0.77 ± 0.21	0.83 ± 0.20	0.75 ± 0.20	**0.03**
**Total dose (mg/kg/60 min)**	0.30 (0.24–0.35)	0.29 (0.24–0.36)	0.30 (0.25–0.35)	0.71
**Number of additional doses**				**0.01**
0	103 (%48)	12 (26%)	91 (54%)	
1	63 (%29)	18 (38%)	45 (26%)	
2	28 (%13)	10 (21%)	18 (11%)	
3	15 (%7)	5 (11%)	10 (6%)	
4	7 (%3)	2 (4%)	5 (3%)	
**Drug used for reversal**				**0.006**
Neostigmine	174 (81%)	45 (96%)	129 (76%)	
Sugammadex	42 (19%)	2 (4%)	40 (24%)	
**Doses of reversal agents**				
Neostigmine (mcg/kg)	21.2 ± 3.0	20.3 ± 2.5	21.5 ± 3.2	**0.02**
Sugammadex (mg/kg)	1.5 ± 0.7	1.1–3.2	2.2 ± 1.5	N/A
**Neuromuscular monitoring**				**0.01**
Yes	78 (%36)	9 (19%)	69 (41%)	
No	138 (%64)	38 (81%)	100 (59%)	
**Reverse-PACU duration (min)**	12.1 ± 3.6	13.6 ± 3.6	11.7 ± 3.5	**0.001**
**NMBD-PACU duration (min)**	104 ± 42	84 ± 35	110 ± 42	**<0.001**

Qualitative data are expressed as number and percentage of case, and compared with chi-squared test. Normally distributed data are expressed as mean ± SD, and compared with Student’s t-test. Nonnormally distributed data are expressed as median (25th to 75th percentile), and compared with the Mann–Whitney U test. Sugammadex dose in RNMB group is expressed as min–max. NMBD: neuromuscular blocking drug, PACU: Postoperative anesthesia care unit. Reverse-PACU duration: Time between the first application of the reversal agent at the end of the surgery and the recording of firs measurements in PACU. NMBD-PACU duration: Time between the application of the last dose of NMBD application and the recording of firs measurements in PACU.

**Table 5:** Multivariable logistic regression analysis for the association between residual neuromuscular blockade and potentially related factors.

	Odds ratio	95% CI	p-value
**Drug used for reversal**			
Sugammadex	1	reference	
Neostigmine	**443.5**	10.7–18355.2	**0.001**
**Use of NMM**			
Yes	1	reference	
No	**3.9**	1.4–10.8	**0.01**
**NMBD-PACU duration (min)**			
145 +	1	reference	
88–144	0.5	0.1–1.6	0.24
51–87	**7.6**	2.2–26.6	**0.001**
0–50	**104.2**	3.3–3300.2	**0.008**
**Additional NMBD doses**			
0	1	reference	
1	1.4	0.5–4.2	0.5
2	**4.4**	1.2–15.5	**0.02**
3	**9.4**	1.3–70.9	**0.03**
4	**112.3**	3.8–3286.6	**0.006**

CI: confidence interval, NMM: neuromuscular monitoring, NMBD: neuromuscular blocking drug, NMBD-PACU duration: Time between the application of the last dose of NMBD application and the recording of firs measurements in PACU.
